# Supplementary material for: A Global Screen for Assembly State Changes of the Mitotic Proteome by SEC-SWATH-MS
Source: Cell Syst. 2020 Feb 26;10(2):133–155.e6. doi: 10.1016/j.cels.2020.01.001 (PMC7042714; doi:10.1016/j.cels.2020.01.001)

O43422 | P52K_HUMAN | THAP12 DAP4 P52RIPK PRKRIR THAP0
Monomer MW [kDa]: 87.704 Monomer expected elution fraction: 41

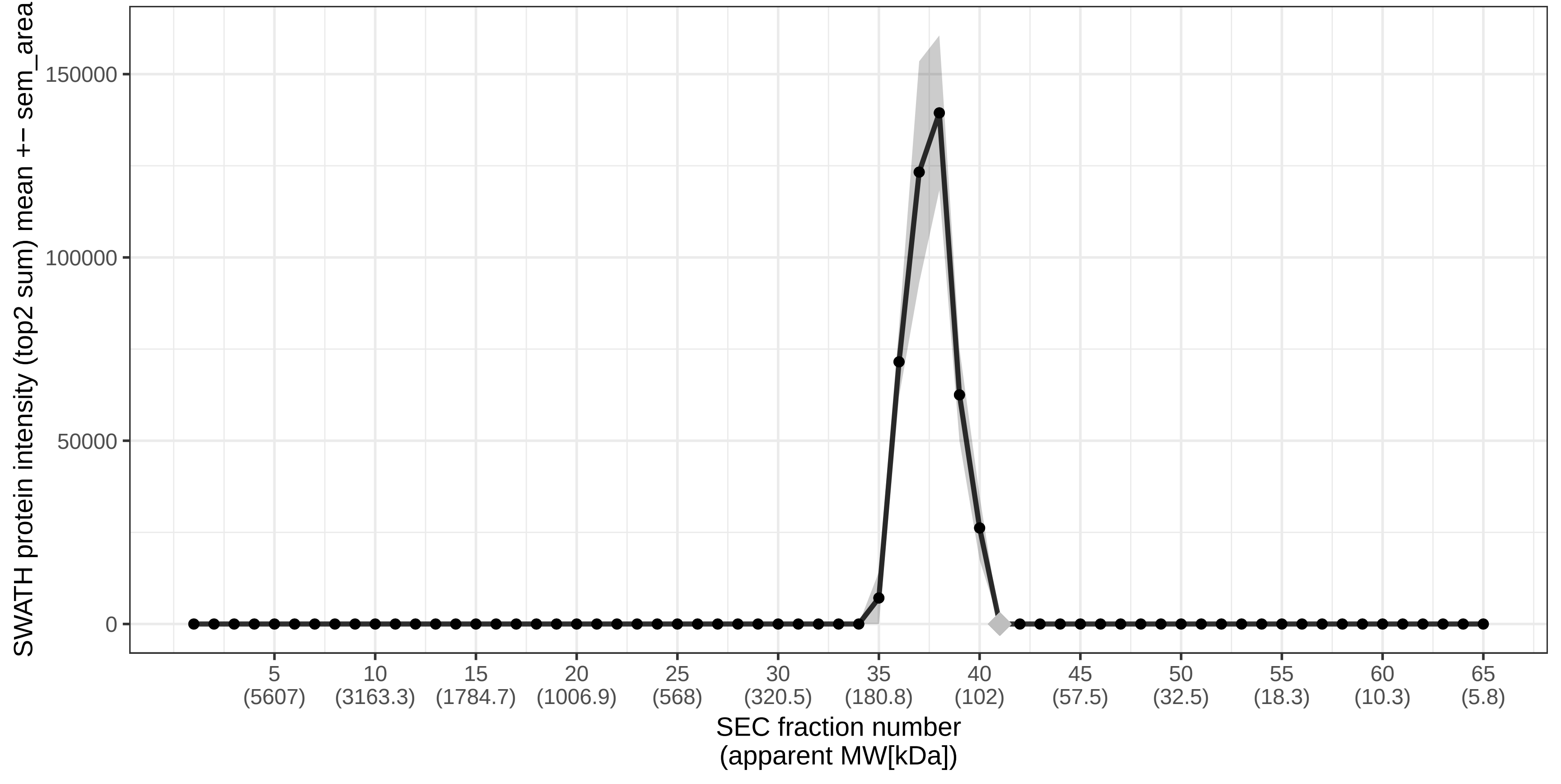

Supplement: Data S1. SEC-SWATH-MS Protein Chromatograms, Related to Figure 1 [file mmc6.zip › SECchrom_O43422_P52K_HUMAN_THAP12_DAP4_P52RIPK_PRKRIR_THAP0.pdf]
